# Supplementary material for: Efficacy of PARP Inhibitor, Platinum, and Immunotherapy in BRCA-Mutated HER2-Negative Breast Cancer Patients: A Systematic Review and Network Meta-Analysis
Source: J Clin Med. 2023 Feb 17;12(4):1588. doi: 10.3390/jcm12041588 (PMC9966507; doi:10.3390/jcm12041588)
Supplement: Supplementary file 1 [file jcm-12-01588-s001.zip › Supplementary MaterialsS1.pdf]

Appendix

Retrieval strategy
Negative results of subgroup analysis
Network structure diagrams of subgroup
Figure S1. Funnel plot of ORR (Egger's test P value = 0.12)
Figure S2. Cochrane Collaboration's tool for assessing risk of bias in randomized trials (RoB2.0)
Figure S3-S11. Forest plots and Node analysis plots based on all incorporated arms of each outcome (containing heterogeneity and consistency results)

Platinum

CENTRAL:
#1 MeSH descriptor Breast Neoplasms explode all trees 218
#2 breast near neoplasm* 16208
#3 breast near carcinoma* 3150
#4 breast near cancer* 39779
#5 breast near tumour* 689
#6 breast near tumor* 3828
#7 #1 or #2 or #3 or #4 or #5 or #6 41944
#8 platinum or cisplatin or cisplatinum or Oxaliplatin or Carboplatin or Lobaplatin 30231
#9 MeSH descriptor Platinum explode all trees 94
#10 MeSH descriptor Cisplatin explode all trees 101
#11 MeSH descriptor Platinum Compounds explode all trees 25
#12 MeSH descriptor Carboplatin explode all trees 70
#13 MeSH descriptor Oxaliplatin explode all trees 36
#14 #8 or #9 or #10 or #11 or #12 or #13 30231
#15 #7 and #14 1704

WHO ICTRP search portal:
Basic searches
1. Platinum-containing regimens for metastatic breast cancer
2. Metastatic breast cancer AND platinum
3. Advanced breast cancer AND platinum
4. Triple negative breast cancer AND platinum
Advanced Searches
1.Condition: metastatic breast cancer OR advanced breast cancer OR triple negative breast cancer Intervention: platinum-containing regime% OR platinum compound% OR platinum% OR cisplatin OR carboplatin OR platin% OR cisplatinum OR carboplatinum OR platinum diamminodichloride OR cis-diamminedicholoroplatinum OR oxaliplatin Recruitment Status: ALL
2. Condition: metastatic breast cancer OR advanced breast cancer OR triple negative

breast cancer Intervention: biocisplatinum OR dichlorodiammineplatinum OR nsc-119875 OR platidiam OR platino OR platinol OR paraplatin OR cis-diamminedichloroplatinum OR cis-platinum OR cyclobutanedicarboxylato OR cbdca OR jn-8 OR nsc-241240 Recruitment Status: ALL

ClinicalTrials.gov:
Basic Searches
1. Condition or disease: Metastatic breast cancer Other terms: platinum 2. Condition or disease: Advanced breast cancer Other terms: platinum 3. Condition or disease: Triple negative breast cancer Other terms: platinum Advanced Searches: 1. Condition or disease: metastatic breast cancer OR advanced breast cancer OR triple negative breast cancer Intervention/treatment: platinum-containing regime% OR platinum compound% OR platinum% OR cisplatin OR carboplatin OR platin% OR cisplatinum OR carboplatinum OR platinum diamminodichloride OR cis-diamminedicholoroplatinum OR oxaliplatin Study type: All Study results: All
2. Condition or disease: metastatic breast cancer OR advanced breast cancer OR triple negative breast cancer Intervention/treatment: biocisplatinum OR dichlorodiammineplatinum OR nsc-119875 OR platidiam OR platino OR platinol OR paraplatin OR cis-diamminedichloroplatinum OR cis-platinum OR cyclobutanedicarboxylato OR cbdca OR jn-8 OR nsc-241240

MEDLINE (via Ovid):
1 randomized controlled trial.pt.
2 controlled clinical trial.pt.
3 randomized.ab.
4 placebo.ab.
5 Clinical Trials as Topic/
6 randomly.ab.
7 trial.ti.
8 (crossover or cross-over).tw. 9 Pragmatic Clinical Trials as Topic/
10 pragmatic clinical trial.pt.
11 or/1-10
12 exp Breast Neoplasms/
13 advanced breast cancer$.tw,sh.
14 advanced breast neoplasm$.tw,sh.
15 advanced breast carcinoma$.tw,sh.
16 advanced breast tumo?r$.tw,sh.
17 metastatic breast cancer$.tw,sh.
18 metastatic breast neoplasm$.tw,sh.
19 metastatic breast carcinoma$.tw,sh.
20 metastatic breast tumo?r$.tw,sh.
21 exp Triple Negative Breast Neoplasms/
22 Triple Negative Breast cancer$.tw,sh.
23 Triple Negative Breast neoplasm$.tw,sh.

24 Triple Negative Breast carcinoma$.tw,sh.
25 Triple Negative Breast tumo?r$.tw,sh.
26 or/12-25
27 exp Cisplatin/
28 exp Carboplatin/
29 cisplatinum.mp.
30 carboplat*.mp.
31 exp Organoplatinum Compounds/
32 exp Platinum/
33 platinum compound*.tw.
34 platinum containing regime*.tw.
35 (platin* or diamminedicholoroplatinum or cis-diamminedichloroplatinum or cis-dichlorodi- ammineplatinum or biocisplatinum or dichlorodiammineplatinum or nsc-119875 or platidiam or paraplatin or cis-platinum or carboplatinum or cyclobutanedicarboxylato or jm-8 or cbdca or nsc-241240).mp.
36 (Carboplatin or Blastocarb or Carboplat or Carboplatin Hexal or Carboplatino or Carbosin or Car- bosol or Carbotec or CBDCA or Displata or Ercar or Nealorin or Novoplatinum or Paraplat or Para- platin AQ or Paraplatin or Paraplatine or Platinwas or Ribocarbo).mp.
37 (Cisplatin or Abiplatin or Blastolem or Briplatin or CACP or CDDP or cis-DDP or cis-diamminedichlo- ridoplatinum or cis-diamminedichloro platinum II or cis-diamminedichloroplatinum or Cis- dichloroammine Platinum II or Cismaplat or Cisplatina or cisplatinous diamine dichloride or cis- platinum II diamine dichloride or cis-platinum II or cis-platinum or Cisplatyl).mp.
38 (Citoplatino or Citosin or CPDD or Cysplatyna or DDP or Lederplatin or Metaplatin or Neoplatin or
PDD or Peyrone's Chloride or Peyrone's Salt or Placis or Platamine or Platiblastin or Platiblastin-S
or Platinex or Platinol-AQ or Platinol or Platinol- AQ VHA Plus or Platinol-AQ or Platinoxan or plat- inum diamminodichloride or Platiran or Platistin or Platosin).mp.
39 (Oxaliplatin or Ai Heng or Aiheng or diaminocyclohexane oxalatoplatinum or oxalatoplatin or oxalatoplatinum or oxaliplatine or Eloxatin or Dacotin or Dacplat or Eloxatine or 1-OHP or L-OHP or oxaliplatin medac).mp.
40 or/27-39
41 11 and 26 and 40
42 Animals/ not humans/
43 41 not 42

Embase (via Ovid):
1 Randomized controlled trial/
2 Controlled clinical study/ 3 Random$.ti,ab. 4 randomization/
5 intermethod comparison/
6 placebo.ti,ab.
7 (compare or compared or comparison).ti.

8 (open adj label).ti,ab.
9 ((double or single or doubly or singly) adj (blind or blinded or blindly)).ti,ab.
10 double blind procedure/
11 parallel group$1.ti,ab.
12 (crossover or cross over).ti,ab.
13 ((assign$ or match or matched or allocation) adj5 (alternate or group$1 or intervention$1 or pa- tient$1 or subject$1 or participant$1)).ti,ab.
14 (assigned or allocated).ti,ab.
15 (controlled adj7 (study or design or trial)).ti,ab.
16 (volunteer or volunteers).ti,ab.
17 trial.ti.
18 or/1-17
19 exp breast cancer/
20 breast cancer$.tw,sh.
21 advanced breast cancer$.tw,sh.
22 advanced breast carcinoma$.tw,sh.
23 advanced breast neoplasm$.tw,sh.
24 advanced breast tumo?r$.tw,sh.
25 exp metastatic breast cancer/
26 metastatic breast cancer$.tw,sh.
27 metastatic breast carcinoma$.tw,sh.
28 metastatic breast neoplasm$.tw,sh.
29 metastatic breast tumo?r$.tw,sh.
30 exp triple negative breast cancer/
31 triple negative breast cancer$.tw,sh.
32 Triple Negative Breast carcinoma$.tw,sh.
33 Triple Negative Breast neoplasm$.tw,sh.
34 Triple Negative Breast tumo?r$.tw,sh.
35 or/21-34
36 (19 or 20) and 35
37 exp cisplatin/
38 exp carboplatin/
39 exp platinum complex/
40 exp platinum/
41 exp oxaliplatin/
42 exp platinum derivative/
43 platinum containing regime*.tw.
44 (platin* or diamminedicholoroplatinum or cis-diamminedichloroplatinum or cis-dichlorodi- ammineplatinum or biocisplatinum or dichlorodiammineplatinum or nsc-119875 or platidiam or paraplatin or cis-platinum or carboplatinum or cyclobutanedicarboxylato or jm-8 or cbdca or nsc-241240).mp.
45 (Carboplatin or Blastocarb or Carboplat or Carboplatin Hexal or Carboplatino or Carbosin or Car- bosol or Carbotec or CBDCA or Displata or Ercar or Nealorin or Novoplatinum or Paraplat or Para- platin AQ or Paraplatin or Paraplatine or Platinwas

or Ribocarbo).mp.
46 (Cisplatin or Abiplatin or Blastolem or Briplatin or CACP or CDDP or cis-DDP or cis-diamminedichlo- ridoplatinum or cis-diamminedichloro platinum II or cis-diamminedichloroplatinum or Cis-dichloroammine Platinum II or Cismaplat or Cisplatina or cisplatinous diamine dichloride or cis- platinum II diamine dichloride or cis-platinum II or cis-platinum or Cisplatyl).mp.
47 (Citoplatino or Citosin or CPDD or Cysplatyna or DDP or Lederplatin or Metaplatin or Neoplatin or
PDD or Peyrone's Chloride or Peyrone's Salt or Placis or Platamine or Platiblastin or Platiblastin-S
or Platinex or Platinol-AQ or Platinol or Platinol- AQ VHA Plus or Platinol-AQ or Platinoxan or plat- inum diamminodichloride or Platiran or Platistin or Platosin).mp.
48 (Oxaliplatin or Ai Heng or Aiheng or diaminocyclohexane oxalatoplatinum or oxalatoplatin or oxalatoplatinum or oxaliplatine or Eloxatin or Dacotin or Dacplat or Eloxatine or 1-OHP or L-OHP or oxaliplatin medac).mp.
49 or/37-48
50 18 and 36 and 49
51 limit 50 to (human and (conference abstracts or embase))
(Continued)

PARP

CENTRAL:
#1 MeSH descriptor Breast Neoplasms explode all trees 218
#2 local* near advance* near breast near cancer* or locally advance* breast cancer* 2094
#3 local* near advance* near breast near neoplasm* or locally advance* breast neoplasm* 947
#4 local* near advance* near breast near carcinom* or locally advance* breast carcinoma* 308
#5 local* near advance* near breast near tumour* or locally advance* breast tumour* 305
#6 local* near advance* near breast near tumor* or locally advance* breast tumor* 952
#7 metasta* near breast near cancer* or metastatic breast cancer* 8710
#8 metasta* near breast near neoplasm* or metastatic breast neoplasm* 3545
#9 metasta* near breast near carcinom* or metastatic breast carcinom* 995
#10 metasta* near breast near tumour* or metastatic breast tumour* 755
#11 metasta* near breast near tumor* or metastatic breast tumor* 3009
#12 #1 or #2 or #3 or #4 or #5 or #6 or #7 or #8 or #9 or #10 or #11 9864
#13 MeSH descriptor Poly(ADP-ribose) Polymerases explode all trees 8
#14 'Poly (ADP-ribose) Polymerase inhibitor' or Poly (ADP-ribose) near Polymerase* near inhibitor 315
#15 PARP* inhibit* or PARP* near inhibit* 808
#16 veliparib or ABT-888 242

#17 olaparib or AZD-2281 722
#18 rucaparib or AG014699 or PF-01367338 or CO-388 142
#19 niraparib or MK-4827 210
#20 talazoparib or BMN673 107
#21 E7449 or E7016 or ABT-767 or BGB-290 or KR-33889 11
#22 #13 or #14 or #15 or #16 or #17 or #18 or #19 or #20 or #21 1523
#23 #12 and #22 192

WHO ICTRP search portal:
Basic search
1. breast cancer AND PARPI
2. breast cancer AND polymerase inhibitor
3. breast cancer AND PARP inhibitor
Advanced search
1.Conditions: Breast cancer* OR breast neoplasm* OR breast carcinoma*
Intervention: PARPI* OR PARP inhibitor* OR polymerase inhibitor*
Recruitment status: All studies
2.Conditions: Breast cancer* OR breast neoplasm* OR breast carcinoma*
Intervention: olaparib OR azd 2281 OR rucaparib OR ag014699 OR pf 01367338 OR co 388 OR veliparib OR abt 888 OR niraparib OR mk 4827 OR talazoparib OR bmn 673 or e7449 OR e7016 OR abt-767 OR bgb-290 or KR-33889
Recruitment status: All studies

ClinicalTrials.gov:
Basic search
1. breast cancer AND PARPI
2. breast cancer AND polymerase inhibitor
3. breast cancer AND PARP inhibitor
Advanced search
1.Conditions: Breast cancer* OR breast neoplasm* OR breast carcinoma*
Interventions: PARPI* OR PARP inhibitor* OR polymerase inhibitor*
Recruitment: All studies
Study type: Interventional studies
2.Conditions: Breast cancer* OR breast neoplasm* OR breast carcinoma*
Interventions: olaparib OR azd 2281 OR rucaparib OR ag014699 OR pf 01367338 OR co 388 OR veliparib OR abt 888 OR niraparib OR mk 4827 OR talazoparib OR bmn 673 or e7449 OR e7016 OR abt-767 OR bgb-290 OR KR-33889
Recruitment: All studies
Study type: Interventional studies

MEDLINE (via Ovid):
1 randomized controlled trial.pt.
2 controlled clinical trial.pt.
3 randomized.ab.

4 placebo.ab.
5 Clinical Trials as Topic/
6 randomly.ab.
7 trial.ti.
8 (crossover or cross-over).tw. 9 Pragmatic Clinical Trials as Topic/
10 pragmatic clinical trial.pt.
11 or/1-10
12 exp Breast Neoplasms/
13 (local$ adj6 advance$ adj6 (breast adj6 (neoplasm$ or cancer$ or carcinoma$ or tumo?r$))).tw.
14 (metasta$ adj6 (breast adj6 (neoplasm$ or cancer$ or carcinoma$ or tumo?r$))).tw.
15 or/12-14
16 exp "Poly(ADP-ribose) Polymerases"/
17 "Poly(ADP-ribose) Polymerase inhibitor".tw.
18 (poly$ adj6 polymerase$ adj6 inhibit$).tw.
19 (poly$ adj6 ribose$ adj6 polymerase$ adj6 inhibit$).tw.
20 (poly$ adj6 ADP$ adj6 polymerase$ adj6 inhibit$).tw.
21 (PARP$ adj6 inhibit$).tw.
22 exp ADP Ribose Transferases/
23 (veliparib or ABT-888).mp.
24 (olaparib or AZD-2281).mp.
25 (rucaparib or AG014699 or PF-01367338 or CO-388).mp.
26 (niraparib or MK-4827).mp.
27 (BMN 673 or E7449 or E7016 or ABT-767 or BGB-290 or KR-33889).mp.
28 or/16-27
29 and/11,15,28
30 animals/ not (humans/ and animals/)
31 29 not 30
32 limit 31 to yr="2008 -Current"

Embase (via Ovid):
1 Randomized controlled trial/
2 Controlled clinical study/ 3 Random$.ti,ab. 4 randomization/
5 intermethod comparison/
6 placebo.ti,ab.
7 (compare or compared or comparison).ti.
8 (open adj label).ti,ab.
9 ((double or single or doubly or singly) adj (blind or blinded or blindly)).ti,ab.
10 double blind procedure/
11 parallel group$1.ti,ab.
12 (crossover or cross over).ti,ab.
13 ((assign$ or match or matched or allocation) adj5 (alternate or group$1 or intervention$1 or pa- tient$1 or subject$1 or participant$1)).ti,ab.
14 (assigned or allocated).ti,ab.

15 (controlled adj7 (study or design or trial)).ti,ab.
16 (volunteer or volunteers).ti,ab.
17 trial.ti.
18 or/1-17
19 exp breast/
20 exp breast disease/
21 (19 or 20) and exp neoplasm/
22 exp breast tumor/
23 exp breast cancer/
24 exp breast carcinoma/
25 (breast$ adj5 (neoplas$ or cancer$ or carcin$ or tumo$ or metasta$ or malig$)).ti,ab.
26 (local$ adj5 advance$ adj5 (breast adj5 (neoplasm$ or cancer$ or carcinoma$ or tumo?r$))).ti,ab.
27 (metastatic adj5 (breast adj5 (neoplasm$ or cancer$ or carcinoma$ or tumo?r$))).ti,ab.
28 or/21-27
29 exp nicotinamide adenine dinucleotide adenosine diphosphate ribosyltransferase/
30 exp nicotinamide adenine dinucleotide adenosine diphosphate ribosyltransferase inhibitor/
31 poly ADP ribose polymerase inhibitor$.mp.
32 (poly adj5 ADP adj5 ribose adj5 polymerase adj5 inhibit$).mp.
33 ((PARP adj5 inhibit$) or PARP inhibit$).mp.
34 exp veliparib/
35 (veliparib or abt-888 or abt 888).mp.
36 exp olaparib/
37 (olaprib or AZD-2281 or AZD 2281).mp.
38 exp rucaparib/
39 (rucaparib or ag014699 or "pf 01367338" or co 388).mp.
40 exp niraparib/
41 (niraparib or MK-4827 or MK 4827).mp.
42 exp talazoparib/
43 (talazoparib or BMN 673 or BMN673).mp.
44 (E7449 or E7016 or ABT-767 or BGB-290 or KR-33889).mp.
45 or/29-44
46 18 and 28 and 45
47 limit 46 to (human and (conference abstracts or embase))
48 limit 47 to yr="2008 -Current"

Negative results of subgroup analysis

ORR of hormone receptor (HR)-positive subgroup (Odds ratios (OR) with 95% CI)

Chemo		
0.46 (0.11, 2.02)	PARPi	
0.078 (0.001, 1.80)	0.16 (0.002, 5.11)	Platinum+Chemo

pCR of hormone receptor (HR)-positive subgroup (Odds ratios (OR) with 95% CI)

Bev+Chemo					
1.06 (0.24, 4.73)	Bev+Chemo+Platinum				
2.32 (0.68, 8.17)	2.18 (0.31, 15.32)	Chemo			
1.41 (0.26, 7.68)	1.33 (0.14, 12.70)	0.61 (0.19, 1.92)	PARPi+Platinum+Chemo		
3.22 (0.52, 20.40)	3.03 (0.29, 32.38)	1.38 (0.36, 5.39)	2.29 (0.39, 13.75)	Platinum	
2.12 (0.38, 12.33)	2.01 (0.20, 19.91)	0.92 (0.26, 3.16)	1.50 (0.48, 4.74)	0.66 (0.10, 4.13)	Platinum+Chemo

DFS of TNBC subgroup (Hazard ratios (HR) with 95% CI)

Bev+Chemo				
1.38 (0.34, 5.59)	Chemo			
2.42 (0.38, 14.33)	1.75 (0.52, 5.52)	PARPi+Platinum+Chemo		
1.97 (0.31, 10.86)	1.43(0.44, 4.13)	0.82 (0.24, 2.67)	Platinum+Chemo	
1.33 (0.09, 20.57)	0.96 (0.04, 20.54)	0.55 (0.02, 15.41)	0.67 (0.03, 18.14)	Bev+Chemo+Platinum

OS of TNBC subgroup (Hazard ratios (HR) with 95% CI)

Platinum+Chemo		
60.17 (0.04, 387.00)	PARPi	
55.31 (0.06, 265.00)	0.92 (0.09, 9.83)	Chemo

75.38 (0.04, 207.39)	1.28 (0.02, 88.91)	1.40 (0.04, 47.82)	Platinum	
77.67 (0.04, 188.21)	1.30 (0.02, 78.02)	1.42 (0.05, 40.28)	1.01 (0.008, 129.70)	Atezo+Chemo

Abbreviations: PARPi for PARP inhibitor, Bev for Bevacizumab, Atezo for Atezolizumab,Chemo for Chemotherapy.

Network structure diagrams of subgroup

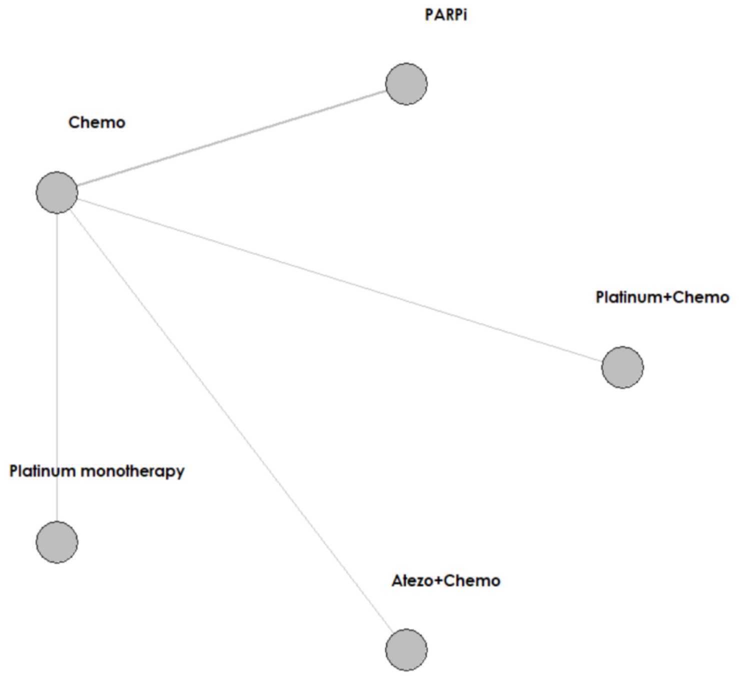

OS of TNBC subgroup

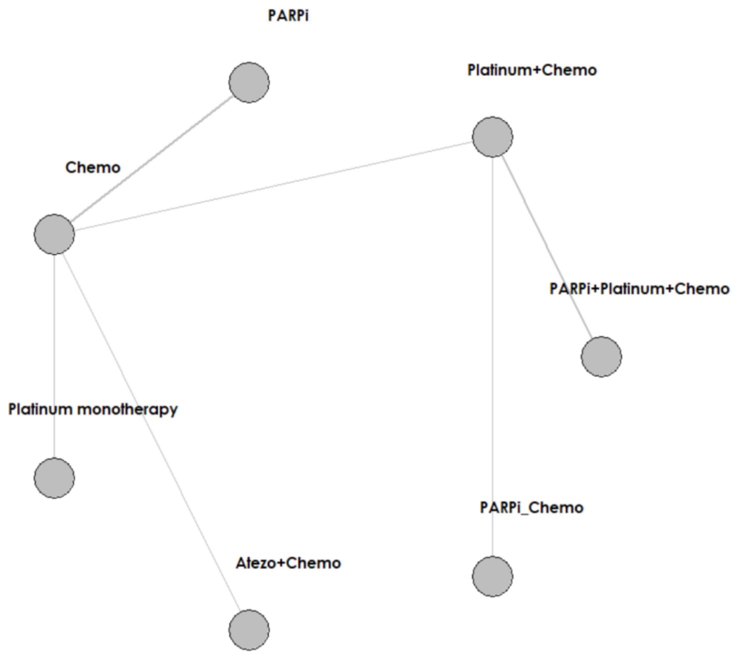

PFS of TNBC subgroup

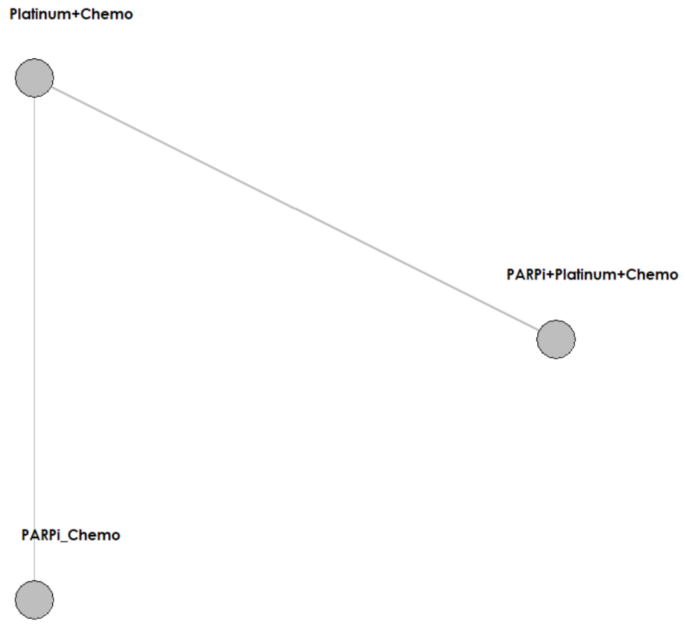

PFS of non-TNBC subgroup

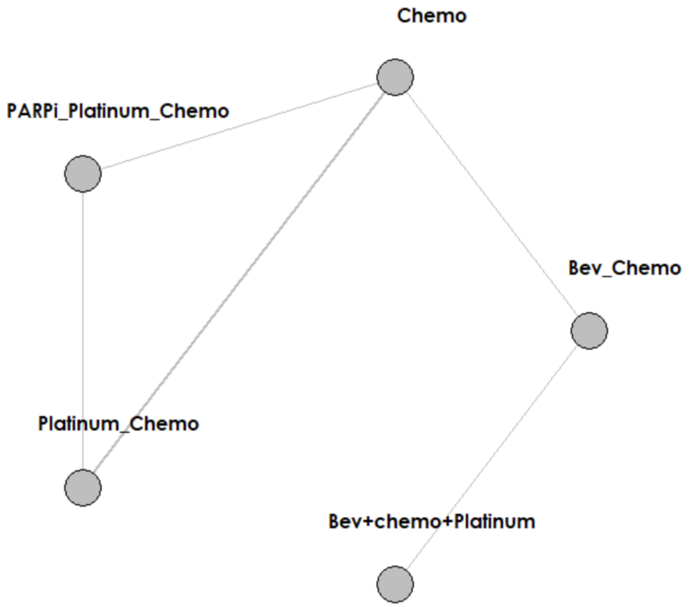

DFS of TNBC subgroup

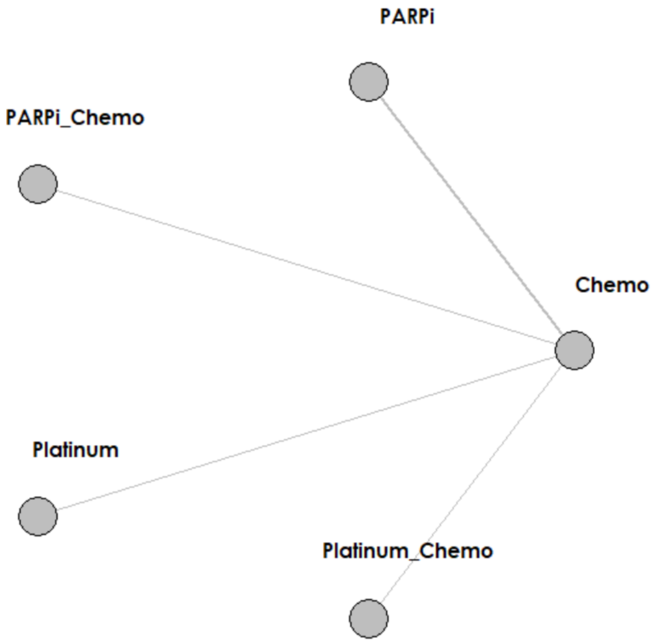

ORR of TNBC subgroup

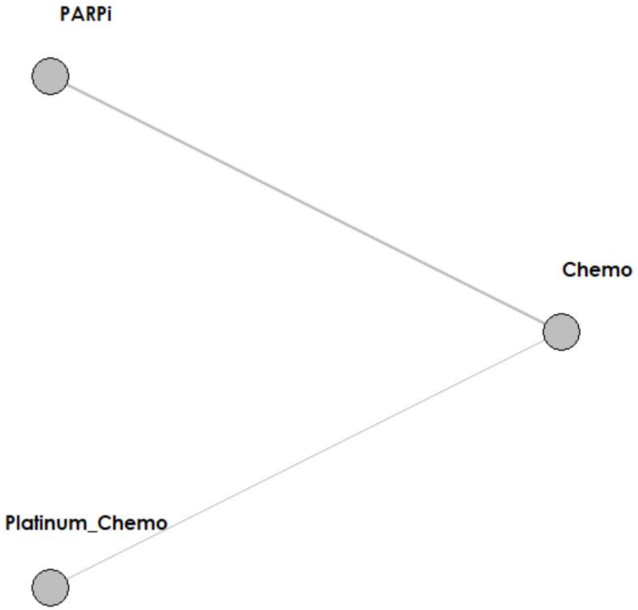

ORR of non-TNBC subgroup

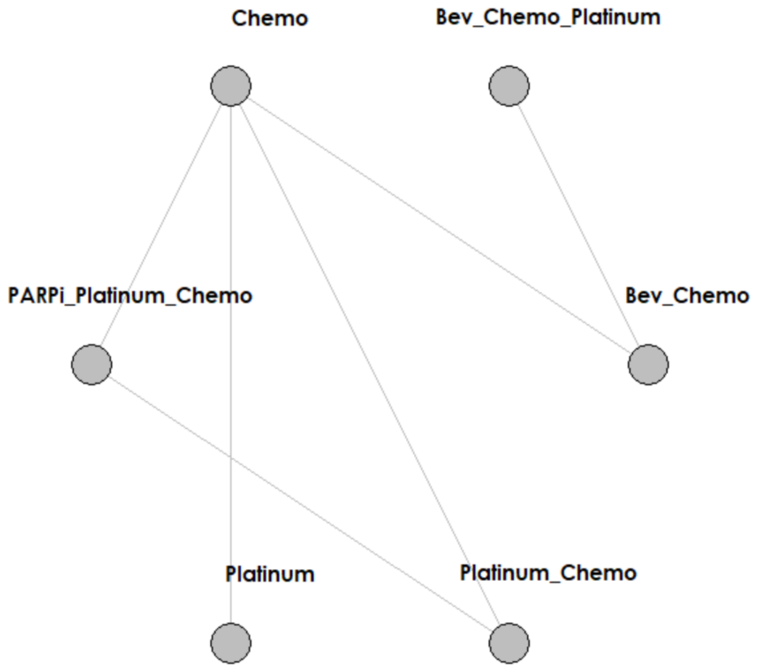

pCR of TNBC subgroup

Abbreviations: PARPi for PARP inhibitor, Bev for Bevacizumab, Atezo for Atezolizumab,Chemo for Chemotherapy.

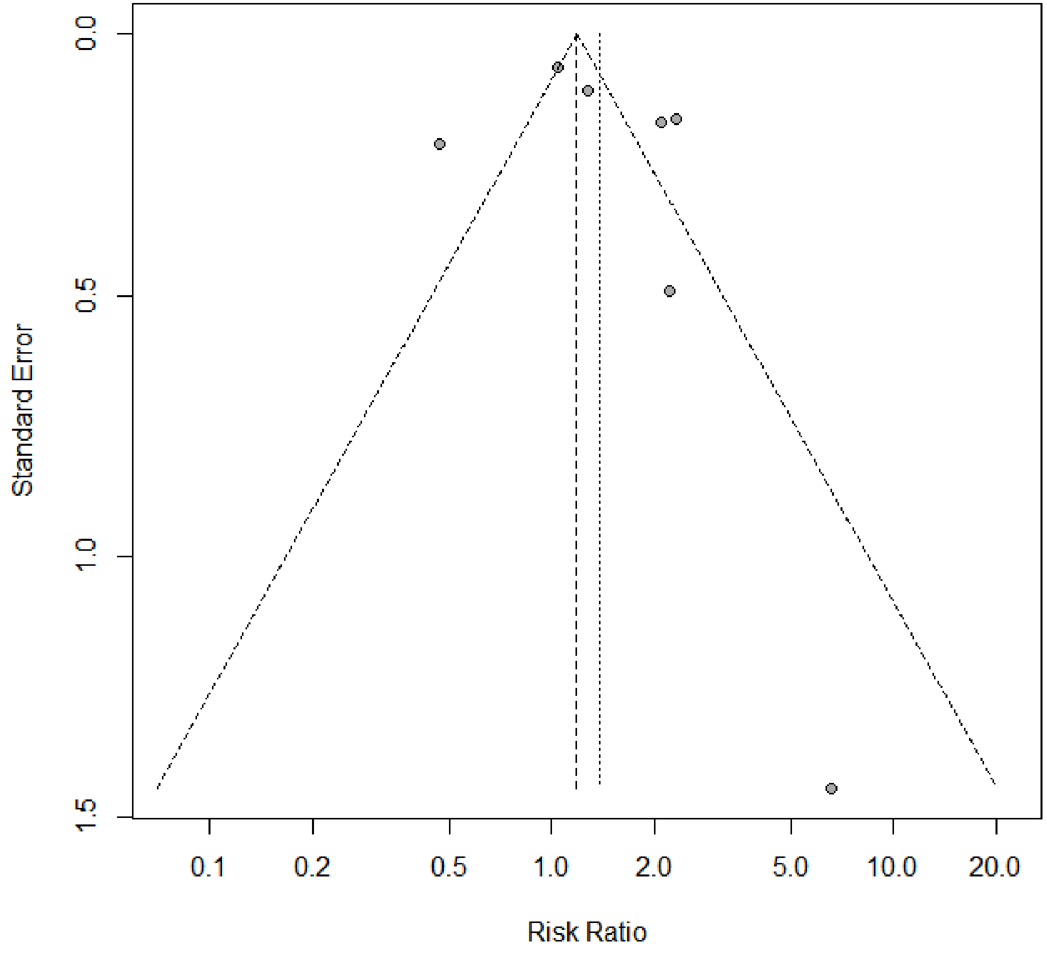

Figure S1 Funnel plot of ORR (Egger's test P value = 0.12)

Color should be used for this figure in print.

Unique ID	Study ID	Randomisation process	Deviations from the intended interventions	Missing outcome data	Measurement of the outcome	Selection of the reported result	Overall
Byrski, T.2010	NA	!	!	!	+	+	!
Fasching P.A. 2021	GeparOLA	+	-	+	+	+	-
Fasching P.A. 2018	GeparQuinto	+	+	+	+	!	+
Geyer, C. E.2022	BrightTNess	+	+	+	+	+	+
Arun, B. K. 2021	BROCADE3	+	+	+	+	+	+
Han, H. S.2018	BROCADE	+	!	+	+	+	+
Hahnen, E.2017	GeparSixto	+	+	+	+	+	+
Robson, M.2019	OlympiAD	+	!	+	+	+	+
Litton, J. K.2020	EMBRACA	+	!	+	+	+	+
Zhang, J.2018	CBCSG006	+	+	+	+	+	+
Pohl, E. 2020	GeparOcto	+	+	+	+	+	+
Tung, N. 2020	INFORM	+	+	+	+	+	+
Turner, N. C.2021	BRAVO	+	!	+	+	+	+
Tutt, A. 2018	TNT	+	!	+	+	+	!
Tutt, A. N. J.2021	OlympiA	+	+	+	+	+	+
Yu, K. D.2020	PATTERN	+	+	+	+	+	+
Emens, L. A.2021	IMpassion130	+	+	+	+	+	+
Du, F. 2020	NA	+	+	+	+	+	+
Kalra, M.2021	NA	+	!	!	+	+	!
Kummar, S.2016	NCT01306032	+	-	+	+	+	-
Zheng, F.2022	NCT01150513	+	+	+	+	+	+
Sella, T.2018	NA	-	+	+	+	-	-

+ Low risk
! Some concerns
- High risk

Figure S2 Cochrane Collaboration's tool for assessing risk of bias in randomized trials （RoB2.0）

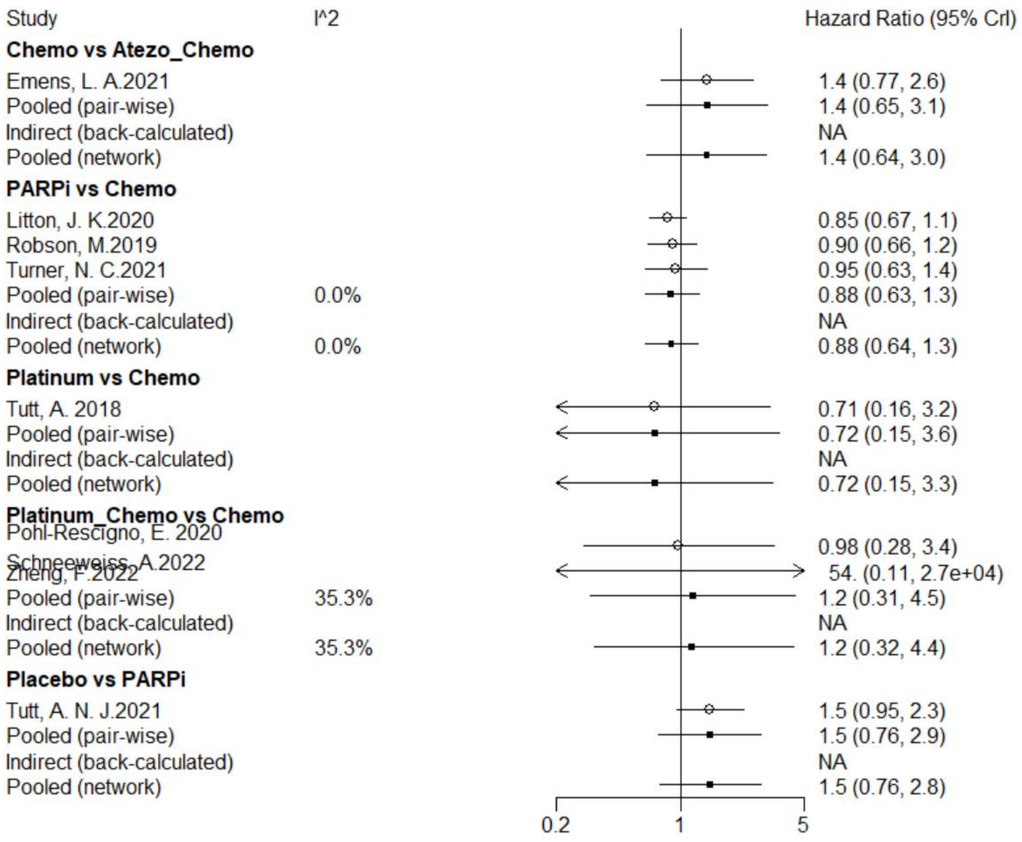

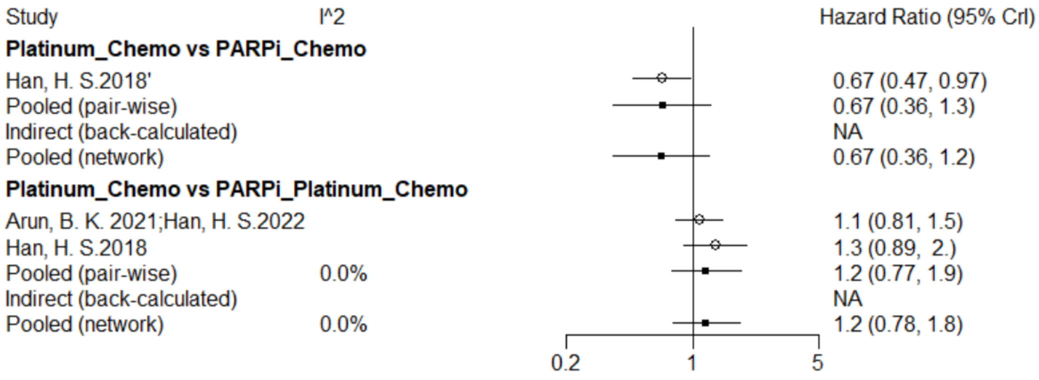

Figure S3 Forest plot of OS. All I^2 were less than 50%. Because of no closed loop, node analysis of consistency could not conduct.

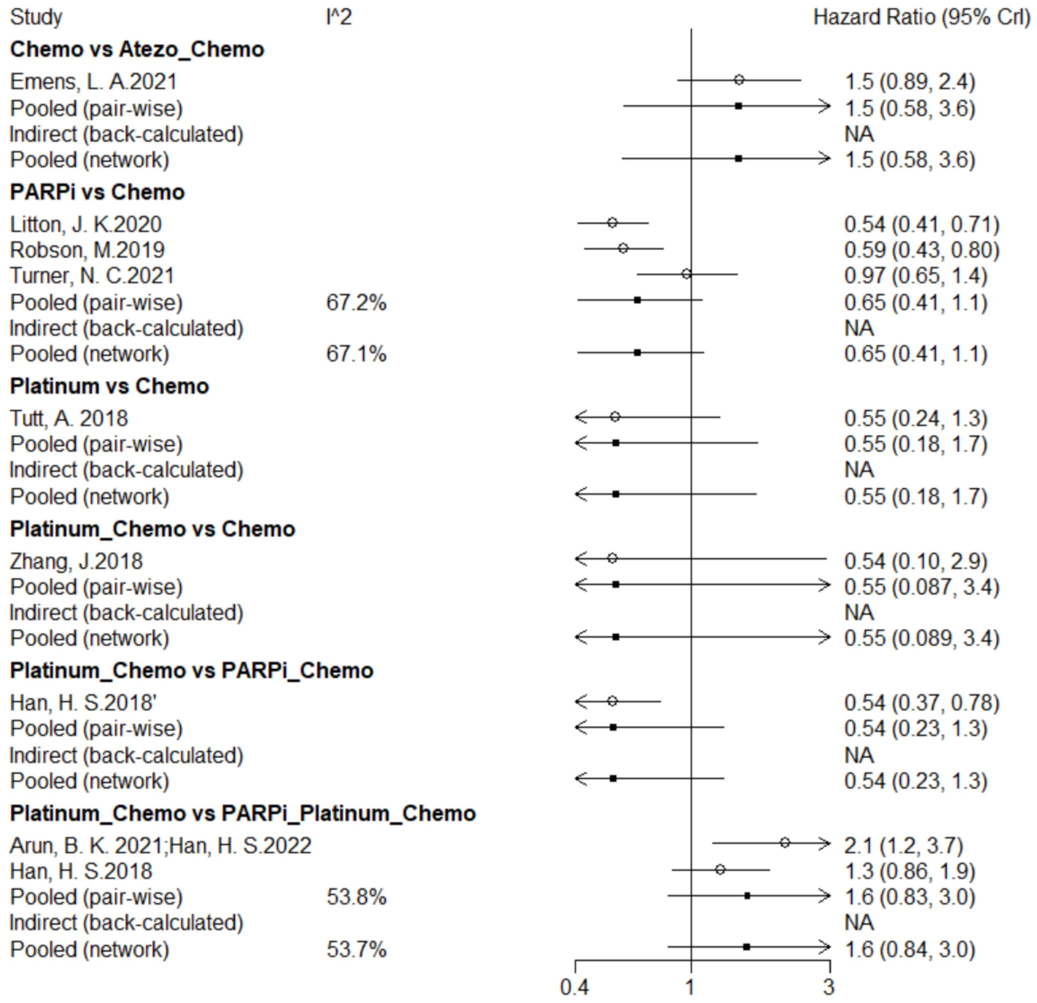

Figure S4 Forest plot of PFS. I^2 were more than 50% in two comparisons (PARPi vs Chemo and Plati-num+Chemo vs PARPi+Platinum+Chemo), which indicated heterogeneity.

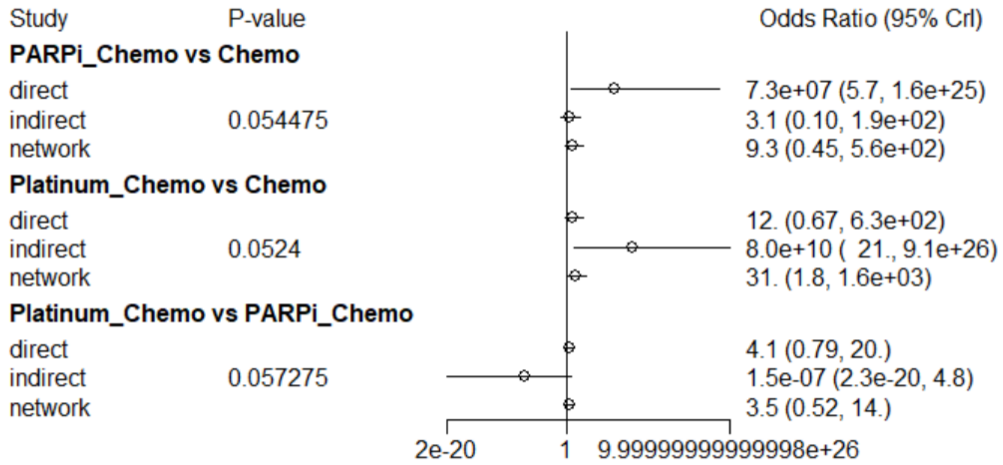

Figure S5 Node analysis of PFS. All P values were more than 0.05, which indicated consistency.

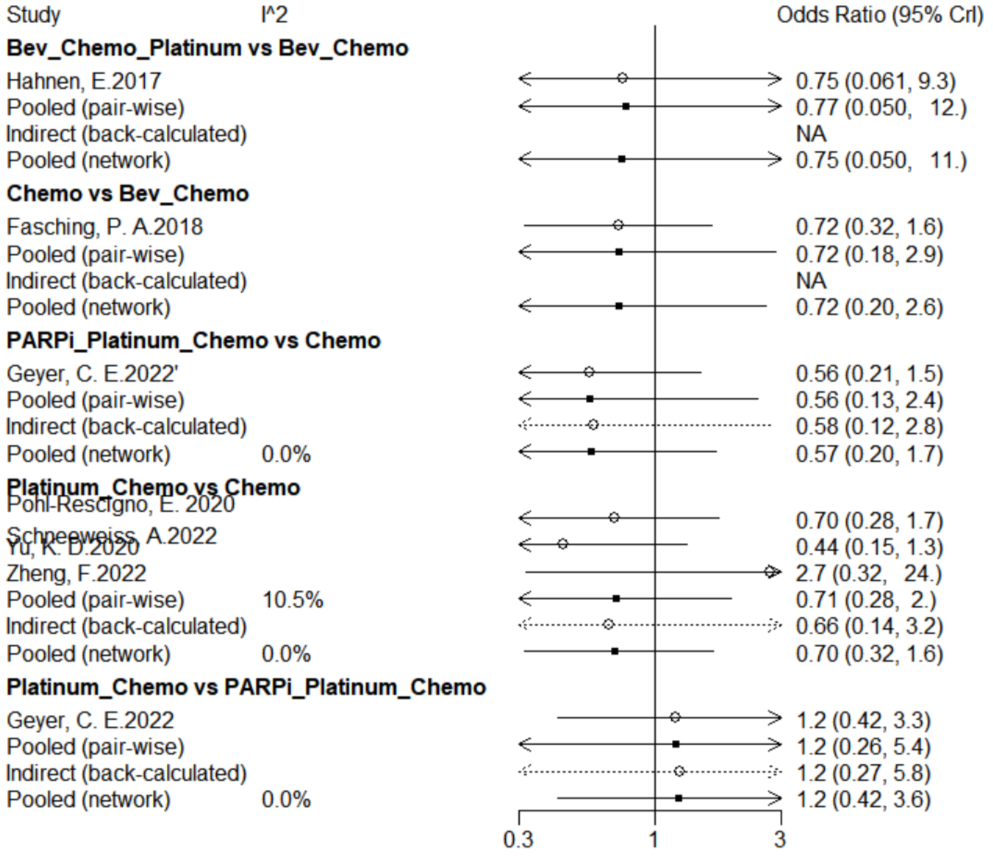

Figure S6 Forest plot of DFS. All I^2 were less than 50%.

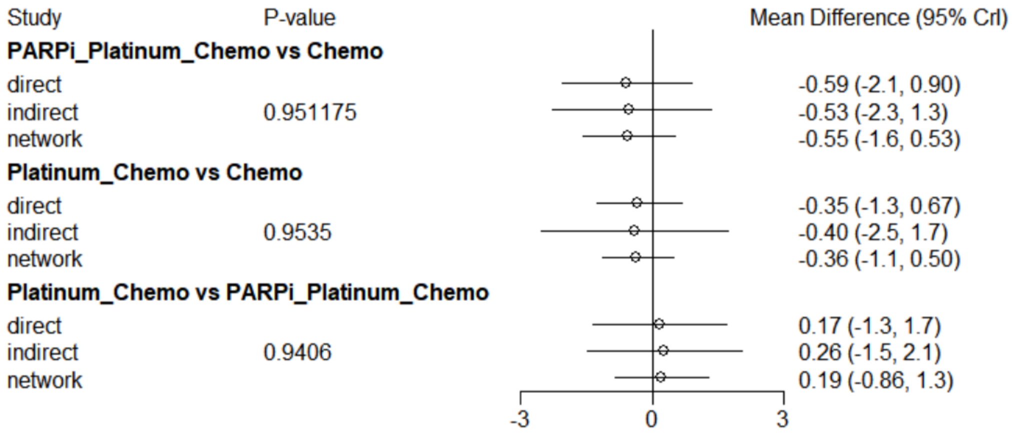

Figure S7 Node analysis of DFS. All P values were more than 0.05, which indicated consistency.

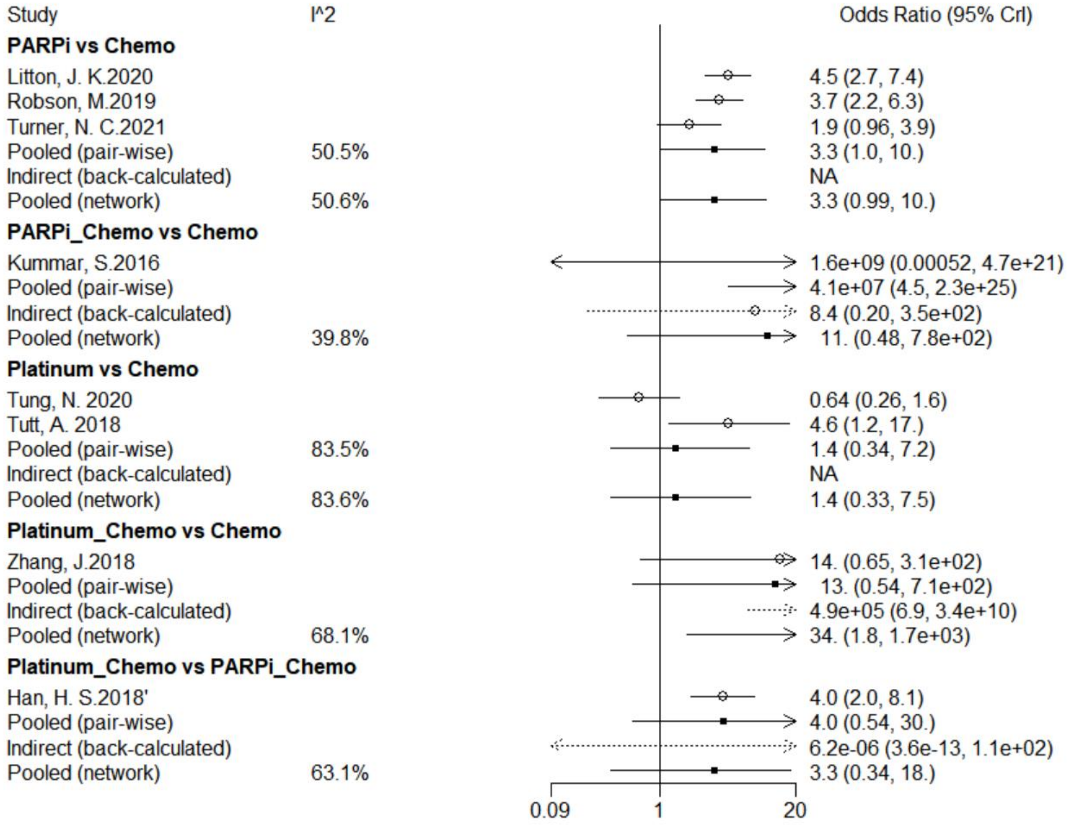

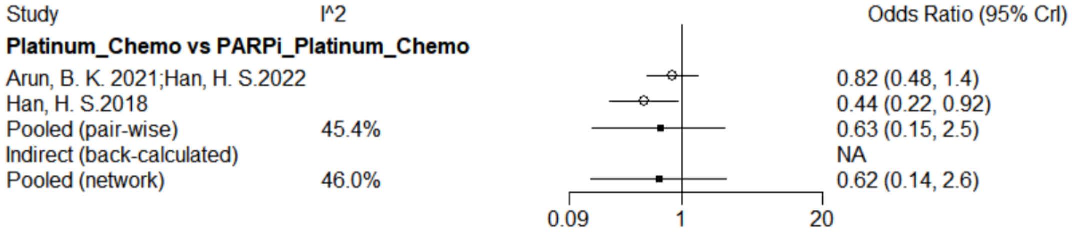

Figure S8 Forest plot of ORR. I^2 were more than 50% in three comparisons (Platinum vs Chemo, Platinum+Chemo vs Chemo, and Platinum+Chemo vs PARPi+Chemo), which indicated heterogeneity.

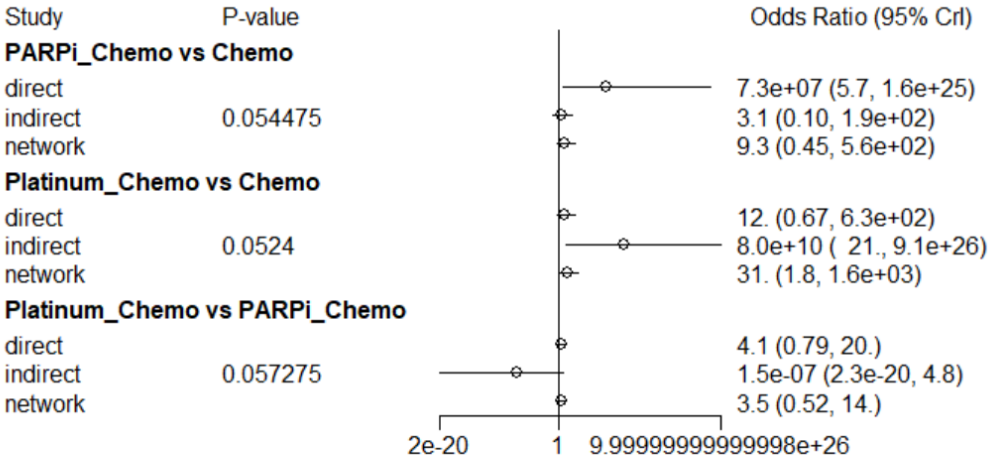

Figure S9 Node analysis of ORR. All *P* values were more than 0.05, which indicated consistency.

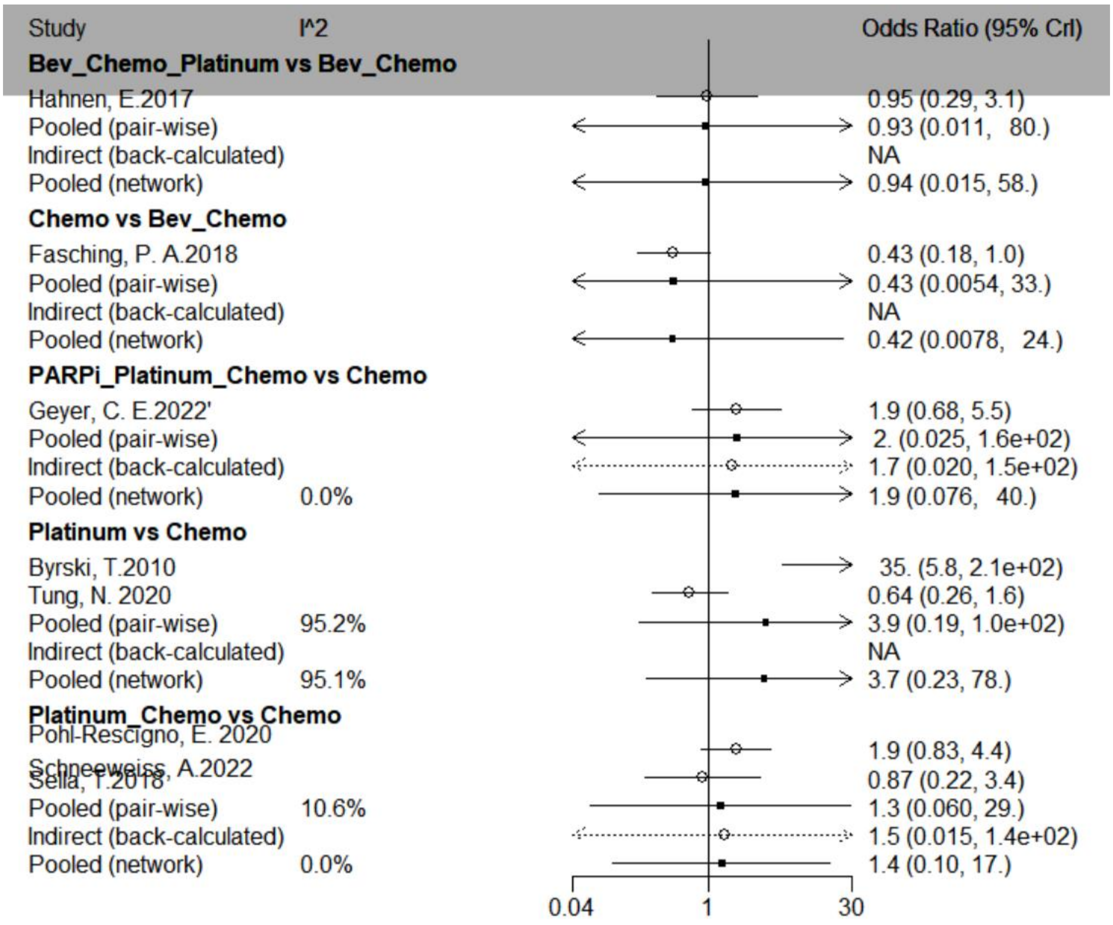

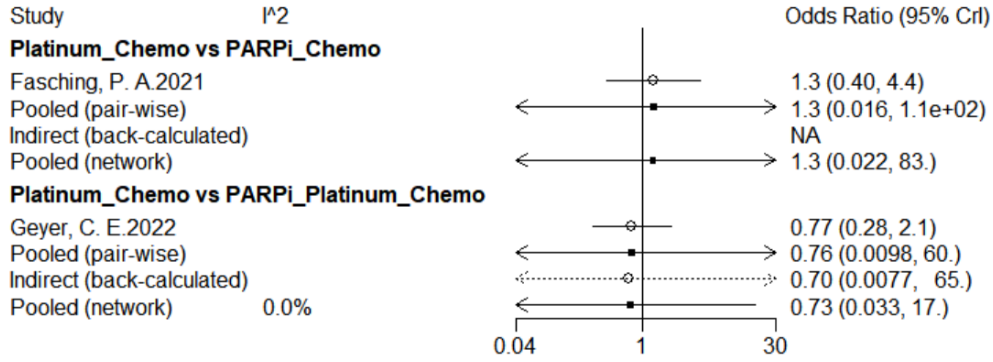

Figure S10 Forest plot of pCR. One group (platinum vs chemo) of I² was more than 50%, which indicated heterogeneity.

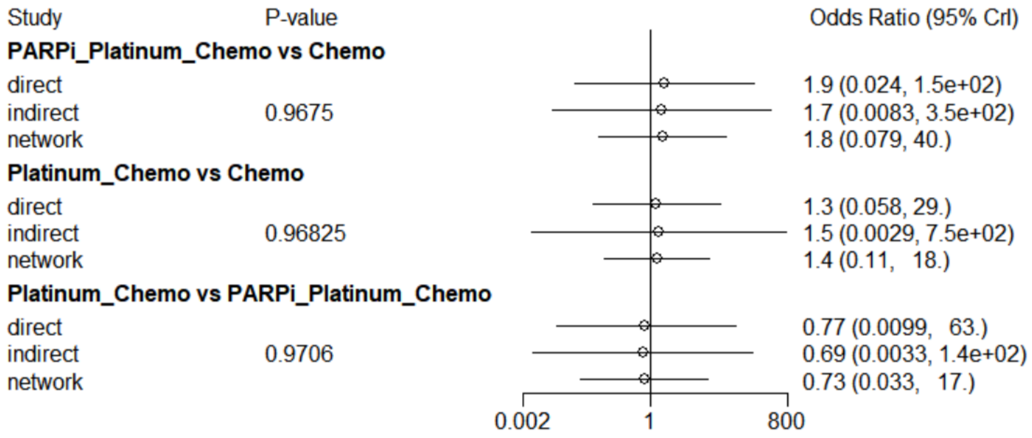

Figure S11 Node analysis of pCR. All *P* values were more than 0.05, which indicated consistency.
